# The Role of Indigenous Epidemic Folk Stories in Shaping Adaptive Disease-Related Behaviors in West Papua

**DOI:** 10.1177/14747049261439652

**Published:** 2026-04-28

**Authors:** Kiriakos Chatzipentidis, Marta Kowal, Piotr Sorokowski

**Affiliations:** 1 Department of Psychology, 49572 University of Wrocław, Wrocław, Poland; 2 IDN Being Human Lab, University of Wrocław, Wrocław, Poland

**Keywords:** storytelling, adaptation, papua, coronavirus disease 2019

## Abstract

Epidemics rank among humanity's deadliest disasters, profoundly affecting societies across health, psychological, social, and economic domains. Past outbreaks have left a unique cultural legacy in the form of epidemic folk stories that explain disease etiology and provide behavioral guidelines during crises. This aligns with theories suggesting that storytelling may serve as an adaptive mechanism facilitating transmission of survival-relevant information. We explored this premise among Indigenous people in West Papua, Indonesia. We interviewed 100 Asmat men, asking them to recall folk stories about past infectious diseases and report their attitudes toward coronavirus disease 2019 (COVID-19). We found that individuals more familiar with epidemic folk stories were more likely to express fear of COVID-19 and adopt social distancing behaviors during the pandemic. Content analysis revealed that these oral narratives emphasized themes such as dietary and good health practices (42%) and hygiene (23%), whereas direct recommendations for social isolation constituted only 12% of identified themes. This pattern suggests that folk stories may not primarily function as precise behavioral instruction manuals, but rather as mechanisms that sensitize individuals to potential disease-related threats. Consistent with this interpretation, exploratory mediation analyses were compatible with the Behavioral Immune System framework, suggesting that folk story knowledge may be associated with heightened sensitivity to pathogen-related cues, stronger emotional responses, and greater behavioral avoidance in threatening contexts. However, the cross-sectional and exploratory nature of our study precludes strong causal or temporal inferences. Longitudinal or experimental research is needed to test these hypotheses and provide stronger evidence for causal interpretation.

Long ago, sickness came to the village. Many died. The people asked the spirits for help. The spirits answered: “Use the natural medicine.” So, they gathered the plants and herbs the spirits told them about. They healed. The village survived.

—Traditional Asmat folk story (West Papua), written down by the authors

## Introduction

Where do stories come from? Why do humans tell stories? Why are stories so powerful? What is the impact of stories on people? Storytelling has been a frequently explored theme for centuries in the humanities, social sciences, and psychology (e.g., Dege & Strasser, 2024; Hutson & Hutson, 2024; Mou, 2024; Nakawake et al., 2024a; Scalise Sugiyama, 2024; Spence, 2024; Wang, 2025). Storytelling, or narratives in general, act as a social and expressive mechanism of culture that engages people and resolves issues and conflicts, creates diversion and enjoyment, and shapes people's understanding of the world (Guenther, 2006). Through the transmission of specific types of information, stories have been even hypothesized to have the power to save the world and its people (Bietti et al., 2019; Boyd, 2009; Gottschall, 2012; Smith et al., 2017; Scalise Sugiyama, 2001).

In this paper, we focus on one of the hypothesized functions of storytelling—the transmission of survival-relevant information (Bietti et al., 2019; Boyd, 2009; Gottschall, 2012; Harari, 2014; Smith et al., 2017; Scalise Sugiyama, 2001). Epidemic folk stories in Indigenous societies may facilitate the transmission of fitness-relevant information, enabling individuals to adopt protective behaviors against biological threats. To test this hypothesis, we conducted a study among the Asmat men in West Papua.

## Epidemics in Human History

Epidemics have inflicted death and socioeconomic catastrophes on civilizations since ancient times (Mahmoudi et al., 2021; WHO, 2022), remaining a global threat (Balloux & van Dorp, 2017) and a persistent feature of human existence (Rainey, 2014). Plague epidemics, possibly the biggest killer in human history (Balloux & van Dorp, 2017), ravaged Europe for over 1400 years (Bramanti et al., 2018), while China experienced 880 epidemic years between 220 BCE and 1949 CE (Lu, 2021). Notable historical epidemics include the Megiddo Plague (1300s BCE) (Rainey, 2014), the Shang Dynasty Plague (1600–1046 BCE) (Liu et al., 2024), the Athenian Plague (430 BCE, estimated fatality rate up to 25%) (Littman, 2009), the Antonine Plague (165–180 CE, fatality rate up to 20%) (Bruun, 2007), the Justinian Plague (541–750 CE, fatality rate 25%–50% of the Roman Empire) (Dattani & Roser, 2023; Mordechai et al., 2019), and the catastrophic Black Death (1346–1353), with fatality rate of 30%–60% in Europe alone (Benedictow, 2004).

Epidemics have intensified since the Middle Ages (Buchy et al., 2021), with dire examples such as the Columbian Exchange (1492–1600, fatality rate 90% of the Indigenous population) (Dattani & Roser, 2023) and the Ming Dynasty plague (1551–1644 CE, death toll of 10 million) (Li et al., 2020). Throughout history, each era has been marked by deadly waves of epidemics that challenged its very existence. The nineteenth century experienced the Seven Cholera Pandemics (1817–present) (Dattani & Roser, 2023; Sampath et al., 2021), the 1889 “Russian flu” (death toll of 4 million) and the Great Bubonic Plague (1894–1940, death toll of 12 million) (Dattani & Roser, 2023; Echenberg, 2007). The twentieth century witnessed devastating influenza pandemics, including the “Spanish flu” (1918, death toll of 50–100 million deaths), the 1957 “Asian flu” (death toll of 2 million), the 1968 “Hong Kong flu” (death toll of 2 million), and the 2009 “Swine flu” (death toll of 1.9 million) (Dattani & Roser, 2023). More recent epidemics, such as HIV/AIDS (1980s–present, death toll of 36 million) (Sampath et al., 2021), SARS, Swine flu, Ebola, and most notably the twenty-first century coronavirus disease 2019 (COVID-19) global pandemic—reinforce the persistent threat of infectious diseases and highlight the critical need for continued research and preparedness efforts to mitigate future outbreaks.

### Impact of Historical Epidemics

Infectious diseases have inflicted more suffering and death than any other natural disaster or war (Mahan et al., 2013), profoundly altering the historical trajectory of humankind (Crawford, 2007). The intertwined history of humanity and pathogenic microbes reveals how cultural shifts from hunter-gatherer lifestyles to agricultural societies and urbanization heightened human vulnerability to infectious agents (Crawford, 2007) and highlights the connection between epidemic diseases and broad cultural and societal transformation, from the earliest plagues, through Black Death to the COVID-19 (Snowden, 2019).

Previous historical pandemics have left an indelible cultural mark on humankind, with the universal plague motif remaining embedded in—and haunting—our collective memory (Carmichael, 1998; Gedi & Elam, 1996), expressed in epidemic myths, legends, and folk stories across cultures (Ranger & Slack, 1992). This enduring motif has inspired works of art across various media, giving rise to popular phrases, paintings, literature, films, and video games (Peiffer-Smadja & Thomas, 2017). Eisenberg and Mordechai (2020) argue that the enduring historical influence of the Justinianic Plague stems not from conclusive evidence of its actual impact but rather from the powerful myth it has become, serving as a warning of the devastating potential of large-scale epidemics. Bubonic plagues, most notably the Black Death, remain the inescapable reference point in any discussion of infectious diseases and their societal impact (Snowden, 2019). In many respects, plague represented the worst imaginable catastrophe, thereby setting the standard against which subsequent epidemics would be judged (Snowden, 2019).

Folklorists have also written extensively on the effects of historical and contemporary oral tradition beliefs about epidemics on human behavior, including comparisons between past and present outbreaks. From a folkloristic perspective, storytelling serves as both a means of disseminating information about contagious diseases and a mechanism for coping with them, functioning as a form of cultural medicine (Kitta, 2019). Indeed, the creation of stories represents human societies’ response to disease outbreaks (Lee, 2014). Decision-making about contamination and contagion is multidimensional and linked to culture, encompassing beliefs and legends manifested in outbreak narratives (Kitta, 2019). Vernacular reactions to COVID-19 illustrate how certain core motifs and texts become activated whenever a new epidemic emerges, with narratives helping to establish the boundaries of danger and safety (Hiiemäe et al., 2021). Crucially, such narratives do not emerge anew with each outbreak—rather they remain in continuous circulation and are recycled opportunistically (Lee, 2014). Understanding cultural responses to epidemic is therefore essential, as Indigenous oral traditions likely preserve valuable knowledge about past outbreaks in the form of stories, which may continue to shape current health-related behaviors (Lee, 2014; Kitta, 2019; Hiiemäe et al., 2021).

### COVID-19 and Its Impact on Societies in Various Domains

The COVID-19 pandemic, a cross-continental outbreak of the SARS-CoV-2 virus transmitted human-to-human, reached its peak from 2019 to 2021, infecting nearly 70% of the global population (Ioannidis, 2022). The World Health Organization estimated a total death toll of approximately 27 million between 2020 and 2023 due to the direct and indirect effects of SARS-CoV-2 (Dattani & Roser, 2023; Taylor, 2022).

COVID-19 spread globally at an extraordinary scale (Komarova et al., 2020). The induced lockdowns and social isolation, reminiscent of science fiction scenarios (Canavan et al., 2020), not only altered people's daily lives (Pietromonaco & Overall, 2021, 2022) but also had profound mid- and long-term consequences for mental health, social dynamics, and economic stability (e.g., Karwowski et al., 2020; Kowal et al., 2020; Sorokowski et al., 2020). These effects highlighted the urgent need for comprehensive support and interventions to address these multifaceted challenges.

The COVID-19 pandemic exposed critical gaps in the capacity to detect, predict, and control novel pathogen outbreaks and highlighted existing disparities in access to affordable healthcare (Sampath et al., 2021). To mitigate the immense burden on healthcare systems, prioritizing pandemic preparedness through increased awareness and proactive measures is crucial (Sampath et al., 2021); one such avenue may be the appreciation of Indigenous oral traditions as repositories of disease-avoidance knowledge (Scalise Sugiyama, 2024).

## Storytelling and Transmitting Survival-Relevant Information

Storytelling, or simply sharing stories (Lauer, 2022), is a universal (Bietti et al., 2019; Boyd, 2009; Gottschall, 2012; Smith et al., 2017) and essential form of human communication (Donahue & Green, 2016), reflecting a uniquely human ability to narrativize information (Lauer, 2022). Across cultures, it takes the form of a collaborative conversational activity focused on producing narrative discourse, where a storyteller narrates a sequence of events (Bietti et al., 2019), facilitating the audience's learning, the sharing of personal memories, and cultural transmission (Bietti et al., 2019; Cappelletti et al., 2004). Stories as sources of information are ubiquitous (Gottschall, 2012) and are expressed in various forms of ritualized, fictional, non-fictional, or everyday conversational narratives (Smith et al., 2017), including gossip, rumors, myths and legends, urban legends, conspiracy theories, scientific facts, branding, personal life events, anecdotes, and jokes (Bietti et al., 2019; Fog et al., 2005; Smith et al., 2017)

### Adaptive Significance of Storytelling

The power of narratives has been recognized for centuries (Donahue & Green, 2016), from the tribes of old to modern societies (Fog et al., 2005). The universal presence of storytelling (Smith et al., 2017) since prehistoric times (George, 2019) and antiquity (Biesele, 1986; Chatzipentidis & Sorokowski, 2025) is consistent with the hypothesis that it may represent an important human adaptation (Bietti et al., 2019; Smith et al., 2017). Humans across cultures (Bietti et al., 2019) tell and listen to stories because they have a natural inclination toward narratives (Boyd, 2009) and need them to understand and communicate with each other (Fog et al., 2005).

A central area of the discussion regarding “Why do humans tell stories?” is the consideration of the potential adaptive significance of stories or of being a storyteller (Bietti et al., 2019; Boyd, 2009; Gottschall, 2012; Pinker, 1997). For instance, research among the Agta hunter-gatherers of the Philippines found that skilled storytellers demonstrate higher reproductive success and that their presence correlates with increased levels of camp-wide cooperation (Smith et al., 2017).

Furthermore, Indigenous oral epidemic knowledge has historically played a crucial role in disease management. For example, during the 1993 Four Corners hantavirus outbreak, Native American Diné elders—drawing from epidemic folk stories—recognized recurring patterns from outbreaks in 1918 and 1933, warning their people about deer mice and recommending burning contaminated clothing and sealing food (Harper & Meyer, 1999; Pottinger, 2005). The transmission of such narratives may be particularly consequential given that fear and behavioral responses to epidemics are interconnected. Research has demonstrated that the evolved psychology of disease-avoidance fear motivates adaptive protective behaviors and positive behavior change (e.g., social distancing, improved hand hygiene), and that fear predicts public health compliance during pandemics (Harper et al., 2021; Schaller et al., 2022). Epidemic folk stories may serve multiple functions by transmitting practical health information, shaping risk perception, maintaining social cohesion, and providing behavioral guidelines during crises, as demonstrated through Goldstein's (2004) analysis of AIDS legends, which revealed how contemporary narratives reflect culturally complex attitudes toward health and illness. This transmission function is not unique to epidemic contexts but may reflect a more fundamental property of storytelling itself. Bietti et al. (2019), in their review of the potential adaptive significance of storytelling, identified the transmission of survival-relevant information as one of its core hypothesized functions.

The foregoing examples illustrate how cultural narratives may interface with biological disease-avoidance mechanisms. Infectious diseases have exerted strong selection pressures on human populations, resulting in both immunological defenses and a complementary Behavioral Immune System—a suite of psychological mechanisms that motivate pre-emptive disease-avoidance behaviors to reduce infection risk before immunological responses are needed (Schaller et al., 2022, Schaller & Park, 2011). This framework provides theoretical grounding for understanding how cultural practices (Schaller, 2006) such as storytelling, which has been employed to communicate public health messages during COVID-19 among Indigenous communities (Martell et al., 2025), may function as psychological mechanisms that promote protective behaviors against infectious threats.

In summary, prior work suggests that storytelling may play a role in transmitting survival-relevant information, functioning as an individual-level cognitive adaptation that guides behavioral responses to environmental hazards. Using data from Asmat men of West Papua, we examined whether higher knowledge of past infectious diseases would predict a more intense emotional response (i.e., higher fear) to the COVID-19 pandemic, as well as greater social distancing during the COVID-19 pandemic. Although the capacity for storytelling is hypothesized to be universal (Bietti et al., 2019; Boyd, 2009; Gottschall, 2012; Smith et al., 2017), we anticipated variation in folk story knowledge due to factors such as individual exposure and cultural transmission fidelity. Capitalizing on this natural variation provides us with the opportunity to examine the link between narrative knowledge and protective behaviors.

## Method

This study was conducted in accordance with the Declaration of Helsinki and approved by the Institutional Ethics Committee of the first author's institution. A two-stage consent process was employed to ensure cultural respect and individual autonomy. First, with the help of a local Indigenous research assistant, we obtained permission from village leaders to conduct research within their community, following local customs. This leadership approval granted access to the community but not consent for individual participation. Second, the research assistant explained the study's purpose, anonymity, and the right to withdraw to each participant, who was recruited by walking through the village. Participants were informed that participation was voluntary and independent of leadership approval. Verbal informed consent was obtained from all participants prior to the interviews. Participants received compensation of 100,000 Indonesian Rupiah (approximately 7 USD).

We also wish to further discuss ethical considerations when conducting research with hard-to-reach populations outside of Western cultural contexts. Specifically, when conducting this research, we prioritized the dignity and agency of local community members, adhered to local customs, and ensured the study operated within a framework of tangible reciprocity. This project contributed to the local economy through the remuneration of Indigenous research assistant and participants, and the acquisition of local resources (travel, accommodation and provisions for researchers). Furthermore, we maintain an ongoing collaborative relationship with the Asmat community and are planning future research projects focusing on Asmat art and culture, representing a long-term partnership commitment.

The study's database is publicly available at OSF (https://osf.io/9re8j/?view_only=821df366fe0f493d9e6f3aef760d929e).

### Participants

The study was conducted in the end of 2023 among the indigenous Asmat men of West Papua, Indonesia (Rockefeller & Gerbrands, 1967; Sorokowski et al., 2024a; 2024b). The study included only male participants. Given the composition of the research team and ethical considerations regarding participant comfort and consent, recruitment was limited to men, who also showed significantly higher willingness to participate in this research context. A total of 100 male participants were recruited from villages in the Agats area, including Uus (*n* = 25), Per (*n* = 25), Amboreb (*n* = 25), and Warse (*n* = 25). The final sample consisted of 87 individuals who reported awareness of the COVID-19 pandemic (23 from Uus, 23 from Per, 24 from Amboreb, and 17 from Warse). The participants’ self-reported ages ranged from 22 to 89 years (*M* = 41.01, *SD* = 14.45). Indonesia as a whole implemented national pandemic control measures (e.g., restrictions on movement, large-scale social restrictions known as PPKM) during COVID-19 (Hansun et al., 2022; Diah, 2020), which theoretically applied to all provinces, including Papua. These measures included limits on gatherings, school closures, and mobility restrictions, but the degree of implementation and communication at the village level (especially in remote regions like Asmat) is not well-documented in available research. To the best of our knowledge, the response to COVID-19 has primarily relied on reduced social contact rather than individual isolation. Guided by missionaries and local leaders, some families temporarily move from densely settled villages to numerous forest camps. Our account is consistent with the reports of other Asmat researchers (Davies, 2020).

### Procedure

#### Data Collection

The data collection process was conducted in close collaboration with an indigenous Papuan research assistant. The assistant played a critical role in executing this project, not merely by providing translation assistance, but also by acting as a cultural guide, facilitating participant recruitment and ensuring that all study protocols and interactions respected local customs. The research assistant was financially remunerated for his expertise and time.

Prior to data collection, we conducted a pilot phase involving unstructured interviews and ethnographic observations with local community members. Based on these conversations, two primary domains emerged with respect to the COVID-19 pandemic: Emotional vigilance (i.e., fear) and behavioral vigilance (i.e., avoiding and self-distancing from others). Consequently, our behavioral items were developed to mirror these local conceptualizations. These two measures align with the Behavioral Immune System framework (Schaller, 2006), which posits that the body's physiological immune system is complemented by a psychological line of defense. This system utilizes aversive emotions, primarily fear and disgust, to detect potential cues of infection risk and motivate proactive avoidance behaviors to minimize contact with pathogens. Additionally, the questions regarding folk story content were designed to test the evolutionary hypothesis that oral narratives may function as a sources of survival-relevant information. This operationalization draws upon the theoretical frameworks of Scalise Sugiyama (2001; 2017), Bietti et al. (2019) and Nakawake et al. (2024b), which propose that narratives may encode solutions to recurrent adaptive problems (e.g., predator avoidance, resource acquisition). Finally, during the piloting phase we also ensured that the research questions were appropriate and culturally sensitive for the local population.

We asked the respondents the following questions:

1. “Did you know that there was a dangerous COVID disease in Papua and in the world 2–3 years ago?”

   Yes – No

2. “Were you afraid of the COVID pandemic?”

Definitely no (1) Rather no (2) - Hard to say (3) - Rather yes (4) - Definitely yes (5)

3. “Have you avoided people outside your village during the COVID-19 pandemic?”

Definitely no (1) Rather no (2) - Hard to say (3) - Rather yes (4) - Definitely yes (5)

4. “Have you tried to keep your distance from others during the COVID pandemic?”

Definitely no (1) Rather no (2) - Hard to say (3) - Rather yes (4) - Definitely yes (5)

5a.“Do you know folk stories of human-to-human infectious diseases that happened in the past when you were a child, or when your parents or grandparents were alive, or even earlier?” Response scale: Definitely no (coded as 1) Rather no (2) - Hard to say (3) - Rather yes (4) - Definitely yes (5)
5b.“Do these folk stories contain recommendations on what to do when facing contagious disease? If yes, what are the MOST IMPORTANT recommendations?”

Items 3 and 4 assessed adherence to social distancing practices (“Have you avoided people outside your village during the COVID-19 pandemic?” and “Have you tried to keep your distance from others during the COVID-19 pandemic?”). Due to their conceptual similarity, these items were treated as a single social distancing subscale. A reliability analysis was conducted to evaluate the internal consistency of this two-item subscale. The analysis yielded a Cronbach's alpha of α = 0.958, demonstrating excellent internal consistency (Cohen et al., 2018). Furthermore, a strong item-total correlation (*r* = 0.920, *p* < 0.05) was observed, indicating that both items effectively measured social distancing.

It is important to note that we did not ask participants whether they had contracted COVID-19. This decision was made because post-hoc verification of infection status would have been unreliable. Additionally, the prevalence of COVID-19 in this region was very low.

#### Definition of Folk Stories

Defining stories presents conceptual challenges, as Stein (1982) argued that “there is not a unique set of features which is used to identify stories” (p. 487), noting that storytelling functions are multifaceted and mirror the wide variety of motives underlying human social behavior. Aristotle's classical definition of *mythos* describes it as constructed around “a single, whole, and complete action, with beginning, middle, and end” (Janko, 1987; Kyriakou, 2024, p. 59). For research involving Indigenous peoples, Bird et al. (2009) advocated for a broader, more culturally appropriate definition of a story as a *sequence* “whereby information is presented as a sequence of connected events, having some kind of thematic or structural coherence” (p. 20).

In the present study, we examined folk stories, defined by Utley (1961) as follows: “So long as the story has had some oral currency we have a folktale” (p. 198) or *folk story* (the terms *folktale* and *folk story* are used interchangeably; Thompson, 1946). All stories reported by the Asmat participants were orally transmitted and therefore qualify as folk narratives. The interviews were conducted with the assistance of a Papuan research assistant.

## Content Analysis of Recommendations from Folk Stories

To analyze responses to question 5b (“Do these folk stories contain recommendations on what to do when facing contagious disease? If yes, what are the MOST IMPORTANT recommendations?”), we employed a content analysis, in which the research team systematically reviewed and transcribed responses to identify behavioral recommendations within epidemic-related folk stories. This analysis was conducted across the full sample (*N* = 100). Responses were examined and categorized based on the research team's collective expertise, including input from our Indigenous Papuan research assistant, to identify recurring themes and patterns in the protective behaviors described by participants. It is important to note that we did not collect or record the complete folk stories themselves; rather, participants were asked to identify and report only the specific health-related recommendations contained within these narratives. This focused approach enabled us to directly capture the most salient health guidance transmitted through oral tradition while respecting the time constraints of the interview context. This methodological decision allowed for the identification of health-protective practices transmitted through Asmat oral tradition without requiring extensive documentation of entire narratives.

We acknowledge that our interpretive framework is shaped by Western academic training and evolutionary behavioral science perspectives, which influenced our hypothesis that Indigenous epidemic folk stories may function as adaptive cultural mechanisms (Berger, 2015). Our categorization of folk story recommendations into biomedical categories (e.g., hygiene, social isolation, traditional medicine) reflects contemporary public health paradigms that may not fully capture the holistic cultural meanings embedded within Asmat oral traditions (Smith, 2012). To partially address these limitations, we collaborated with a local Papuan research assistant.

## Statistical Analysis

In the first step, the distribution of the variables of interest (i.e., folk story knowledge, fear of COVID-19, and social distancing behaviors during COVID-19 pandemic) was investigated. Then, bivariate relationships between these three variables were examined using Spearman's rank correlation coefficients. Next, the frequency distributions for the thematic categories identified within the folk narratives were calculated.

To test whether folk stories may be related to more severe behavioral protective responses during challenging times through higher susceptibility to fear, a mediation analysis with a maximum likelihood estimator was conducted. In this model, folk story knowledge served as the predictor, fear of COVID-19 as the mediator, and social distancing behaviors as the outcome. All analyses were conducted in R (Version 4.5.1).

## Results

Inspection of the frequency distributions of fear, avoidance, and distancing items revealed that they were heavily concentrated at the scale endpoints (options 1 and 5), with very few responses at option 2 across all three variables (see Table 1). The folk story knowledge variable showed a similar pattern, though less symmetrically bimodal, with a strong concentration at options 5 and 3. The nature of these distributions is addressed further in the Limitations section.

**Table 1.** Response Frequency Distributions (Counts and Percentages) for the Four Variables of Interest.

Response Option	Folk Story Knowledge	Fear of Coronavirus Disease 2019 (COVID-19)	Avoidance^a^	Keeping Distance^a^
1 - Definitely no	1 (1.1%)	20 (23.0%)	28 (32.2%)	30 (34.5%)
2 - Rather no	1 (1.1%)	2 (2.3%)	1 (1.1%)	1 (1.1%)
3 - Hard to say	16 (18.4%)	7 (8.0%)	7 (8.0%)	4 (4.6%)
4 - Rather yes	11 (12.6%)	9 (10.3%)	14 (16.1%)	11 (12.6%)
5 - Definitely yes	58 (66.7%)	49 (56.3%)	37 (42.5%)	41 (47.1%)

**^a^** The two variables were combined and averaged in subsequent analyses.

Spearman correlation analyses revealed significant positive associations among the three variables of interest. Folk story knowledge was positively correlated with both fear of COVID-19 (*ρ* = 0.381, *p* < 0.001) and social distancing behaviors (*ρ* = 0.244, *p* = 0.023). Additionally, a strong positive correlation was observed between fear of COVID-19 and social distancing behaviors (*ρ* = 0.558, *p* < 0.001).

 Table 2 presents the frequency of specific recommendations identified in the content analysis of the folk stories. The most frequently mentioned themes were dietary practices and hygiene practices. Notably, direct recommendations for “isolation from other people” constituted only 12% of the identified themes. This suggests that while general folk story knowledge predicts social distancing, the specific instruction to isolate is not a predominant recommendation of these narratives.

**Table 2.** The Frequency Distributions (Counts and Percentages) of Each Theme Identified in the Content Analysis.

Theme	Frequency
Basic dietary and good health practices (e.g., “full eat/drink,” “eat boilwater good food”)	42 (42%)
Hygiene practices (e.g., “not play mud,” “always clean”)	22 (22%)
Traditional medicine (e.g., “drink herbs”)	12 (12%)
Isolation (e.g., “no go anywhere,” “avoid from outsiders”)	12 (12%)
General caution (e.g., “be careful”)	8 (8%)
Unusable (“don’t know”/no response)	4 (4%)
Spiritual and supernatural practices (e.g., “pray to the Lord”)	1 (1%)

The content analysis suggested that folk stories may not function as technical instruction “manuals” that align perfectly with modern medical recommendations. Therefore, we exploratorily tested an alternative hypothesis that such stories function as “cultural danger cues” that activate the behavioral immune system (Schaller, 2006). To examine this, we performed a mediation analysis with social isolation during the COVID-19 pandemic as an outcome variable, folk story knowledge as a predictor, and fear of the COVID-19 pandemic as a mediator.

We want to highlight that our primary causal target of inference in the mediation analysis is the effect of folk story knowledge on social distancing behavior during COVID-19, and whether this effect operates through heightened fear. Identifying this causal effect from cross-sectional observational data requires assumptions, including, most critically, the absence of unmeasured confounding and the correct temporal ordering of variables, which cannot be verified in the present study (Rohrer, 2018). We nonetheless present these estimates and interpret them cautiously, as the proposed relationships are theoretically plausible and therefore potentially meaningful. However, more studies directly testing this hypothesis are needed to establish stronger evidence for causality.

**Figure 1.**
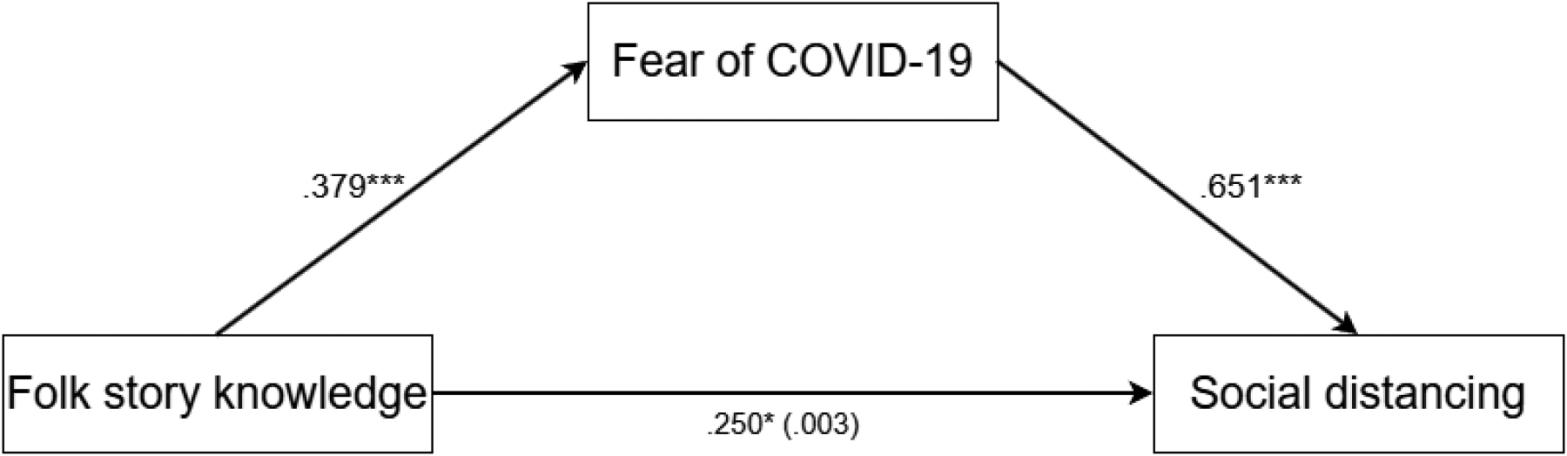
A visual representation of the mediation model (standardized coefficients) with the outcome variable being social distancing during coronavirus disease 2019 (COVID-19), the predictor variable folk story knowledge, and the mediator variable fear of COVID-19.

*Note.* *p < 0.05, **p < 0.01, ***p < 0.001.

The results of this mediation model are consistent with a pattern of full mediation. The association between folk story knowledge and social distancing behaviors was no longer significant after introducing the mediator (fear of the COVID-19 pandemic). Greater knowledge of folk stories was associated with heightened fear of the pandemic, which in turn was strongly associated with increased adherence to social distancing measures (see Table 3 and Figure 1 for detailed results).

**Table 3.** A Summary of the Mediation Analysis.

Path	B	*SE*	95% CI	*p*
Folk story knowledge -> Fear of coronavirus disease 2019 (COVID-19) (path a)	0.693	0.182	[0.337,1.048]	<0.001***
Fear of COVID-19 -> Social distancing (path b)	0.682	0.092	[0.502,0.863]	<0.001***
Folk story knowledge -> Social distancing (direct effect)	0.006	0.168	[-0.324,0.336]	0.971
Indirect effect (path a* b)	0.472	0.139	[0.2,0.745]	<0.001***
Folk story knowledge -> Social distancing (total effect)	0.479	0.199	[0.089,0.869]	0.016*

Note. *p < 0.05, ** *p* < 0.01, ***p < 0.001.

To address potential ambiguity in the response option “3—hard to say” (which may have been understood more as “I don’t know” than “neither yes nor no”), we re-ran the analyses excluding data from eight individuals who selected this option for any of the two social distancing measures. The pattern of results remained exactly the same (see Tables S1 and S2 in Supplemental Materials).

Finally, we conducted a series of additional robustness checks. First, we controlled for two potential confounders: Age and Village, entered into two separate models to avoid overfitting in a model with *N* = 87 (Babyak, 2004). As indicated by the bivariate correlations, Age was unrelated to any of the three variables of interest and controlling for Age did not change the pattern of mediation results (see Tables S3 and S4 in the Supplemental Materials). There were, however, significant differences in social distancing behaviors across Villages, but controlling for this factor did not change the pattern of results in the mediation model (see Table S5 in the Supplemental Materials). Second, we re-ran the mediation model treating variables of interest as categorical rather than continuous variables (collapsed as low [1–2], moderate [3], and high [4–5], given the distributional clustering), for each distancing item separately (avoiding the need to treat the composite as a continuous variable) and using the weighted least squares mean and variance-adjusted estimator. Again, the results remained the same (see Tables S6–S7 in the Supplemental Materials). In summary, all these additional tests yield consistent results with the main mediation analysis (Table 3).

## Discussion

In this study, we explored the relationship between knowledge of folk stories about previous infectious diseases and pandemic-related emotions and behaviors among Asmat men of West Papua. In doing so, we sought to shed further empirical light on whether storytelling may be associated with the transmission of survival-relevant information. The results yielded mixed support for such predictions. On the one hand, knowledge of traditional folk stories was linked to social distancing behaviors during the COVID-19 pandemic. On the other hand, content analysis of the recommendations from stories recalled by participants indicated various potential strategies during outbreaks of human-to-human infectious diseases, with only 12% of recommendations including some form of social isolation. This suggests that stories participants recalled did not act as precise behavioral instruction manuals aligned with modern medical recommendations (e.g., do *X* to survive *Y*). Instead, the results of an exploratory mediation analysis were consistent with an interpretation in which folk stories may act as cultural danger cues that are compatible with the activation of the behavioral immune system (Schaller, 2006). Specifically, we observed that greater folk story knowledge was linked to higher fear of the COVID-19 pandemic, and that fear was strongly associated with increased adherence to social isolation. Importantly, the association between folk story knowledge and social distancing became non-significant when controlling for emotional fear (the mediator), a pattern consistent with full mediation.

Although, our cross-sectional design precludes strong causal conclusions (Maxwell & Cole, 2007), the results are at least tentatively consistent with a theoretically motivated causal pathway. Specifically, knowledge of epidemic folk stories may heighten sensitivity to disease-related threats, intensifying fear, which could in turn motivate protective behavioral avoidance. The suggested mechanism is plausible within the Behavioral Immune System framework (Schaller & Park, 2011), which posits that psychological mechanisms detect pathogen-related cues and trigger aversive emotional and behavioral responses, and is consistent with prior evidence that fear functions as a proximal motivator of protective behavior during outbreaks (Harper et al., 2021).

We want to note that, although plausible and theoretically meaningful (Hernán, 2018), this causal interpretation must remain tentative. Our cross-sectional design does not permit verification of temporal ordering (Maxwell & Cole, 2007), and several alternative explanations cannot be ruled out. For instance, reverse causality is also plausible. Individuals already prone to higher dispositional fear may be more likely to selectively attend to and encode epidemic narratives rather than such narratives causing heightened fear. Additionally, unmeasured third variables, such as trait neuroticism or general health anxiety, could inflate associations across all three variables simultaneously, producing a statistical pattern consistent with mediation even if the true causal structure differs from the proposed pathway. More longitudinal and experimental studies are needed to evaluate the proposed mechanism with greater certainty.

Our findings are in line with prior descriptions of Indigenous knowledge as an important source of resilience and health-related practices, and they corroborate with work that highlights the role of oral traditions in transmitting community experiences (Iseke, 2013; O'Keefe et al., 2021; Petrov et al., 2021; Zibengwa et al., 2021). Indigenous communities, including the Asmat, possess a deep collective memory of historical pandemics shaped by experiences combating infectious diseases such as smallpox and tuberculosis (O'Keefe et al., 2021), leaving lasting ancestral memories and fears of mortality that inform contemporary understandings of disease. Petrov et al. (2021) suggested that this cultural memory may have offered a protective advantage to Indigenous-dominant Arctic communities during the 1918 influenza pandemic. Evidence from other regions further illustrates this pattern. During the COVID-19 pandemic, Indigenous communities in Northeast India enacted formalized isolation rituals rooted in oral tradition. The Kuki people of Manipur performed the Aikam ceremony, triggering a 15-day village lockdown, while Tani tribes of Arunachal Pradesh employed customary isolation lasting 2–15 days, in some cases erecting bamboo gates to barricade villages (Ghosh, 2020; Haokip, 2022). In the Amazon, the Yanomami retreated deeper into the forest, and the Shawi and Ashaninka peoples, drawing on memories of past measles outbreaks, sought forest refuge (Pickering et al., 2023; Zavaleta-Cortijo et al., 2023). Among the Jaleswar tribe of Odisha, lower COVID-19 caseloads were partly attributed to existing social distancing practices aligned with public health recommendations (Sanyal et al., 2022). Notably, while self-imposed isolation was the most common COVID-19 strategy among Indigenous communities globally, reported in approximately 31% of cases reviewed by Pickering et al. (2023), explicit isolation recommendations constituted only 12% of the folk story content in our sample, consistent with our interpretation that these narratives function as sensitizing agents rather than precise behavioral guides. Together, these findings suggest that traditional ecological knowledge transmitted through oral tradition may complement modern public health measures by providing a cultural framework that aligns with protective behaviors during outbreaks.

Despite descriptions of traditional preventive measures (Ghosh, 2020; Sanyal et al., 2022; Zavaleta-Cortijo et al., 2023), the underlying rationale and effectiveness of these practices remain unclear (Gumbo & Gaotlhobogwe, 2021; Petrov et al., 2021). This lack of clarity, coupled with limited research on the impact of COVID-19 on traditional communities (Pickering et al., 2023), highlights a gap in understanding the interplay between Indigenous knowledge and pandemic-related behaviors. The present study contributes to addressing this gap by examining a plausible mechanism through which storytelling may contribute to adaptive responses. The results provided evidence that folk stories about previous infectious diseases were associated with higher fear, which, in turn, was associated with greater social distancing. The exploratory mediation findings align with the behavioral immune response framework, which suggests that certain environmental cues may trigger avoidant behaviors (Schaller, 2006) and with research on fear as a motivating factor in preventing self-harm and promoting adaptive behaviors (Kemp et al., 2021; Presti et al., 2020; Ruiter et al., 2014). Prior research also suggests that the human cognitive system may be “tuned” to retain information relevant to survival (Nairne et al., 2007), such as knowledge about pathogens and ancestral disease narratives. Although the proposition that cultural narratives and Indigenous knowledge may shape behavioral responses during outbreaks is compelling, our study should be treated as preliminary. Accordingly, future studies, preferably using experimental designs, should empirically verify these findings, as our study does not allow for drawing strong or definitive causal inferences.

Content analysis of the folk stories in our study further show that Indigenous epidemic narratives emphasize measures similar to World Health Organization non-pharmaceutical intervention guidelines, including social distancing, nutritious diet, and hygiene (WHO, 2019). Specifically, Asmat folk stories highlighted dietary and hygiene practices, traditional ethnomedicine, and social distancing. Taken together, these results suggest that epidemic storytelling, as a medium for transmitting Indigenous knowledge, may play an important role in health crises management. More broadly, our findings align with existing research showing that Indigenous communities relied heavily on traditional ecological knowledge—such as ethnomedicine—during the COVID-19 pandemic (Johannes, 1989; Sanyal et al., 2022), which is passed down orally and integrates plant-based remedies, spiritual practices, and environmental factors into healing practices (Gumbo & Gaotlhobogwe, 2021; Ghosh, 2020; Pickering et al., 2023; Sanyal et al., 2022; Zavaleta-Cortijo et al., 2023; Zibengwa et al., 2021).

Ultimately, our findings contribute to the literature suggesting that storytelling is not merely a cultural artifact but also an adaptive mechanism that may enhance individual responsiveness to environmental threats (Bietti et al., 2019; Smith et al., 2017; Scalise Sugiyama, 2001). The transmission of survival-relevant information encompasses passing down the knowledge and critical details necessary for navigating adaptive challenges, particularly under demanding conditions, across generations (Bietti et al., 2019; Smith et al., 2017; Scalise Sugiyama, 2001). This is consistent with storytelling's unique properties, which, in ancestral settings, may serve as a mechanism for acquiring, storing (Scalise Sugiyama, 2001), and disseminating survival-relevant information. Our research offers preliminary evidence consistent with the hypothesis that storytelling can, indeed, transmit survival-relevant knowledge.

Although our study provides insights into the potential adaptive value of epidemic storytelling among the Asmat men of West Papua, several limitations should be noted. First, the sample consisted exclusively of male participants and was not representative of the Asmat population, which limits the generalizability of the findings to the broader Asmat group. Future research should include more diverse samples to explore potential gender differences in the links between folk story knowledge and protective behaviors.

Second, the study focused on a single cultural group, the Asmat, which restricts the applicability of the results to other indigenous populations. Cross-cultural studies are needed to determine the extent to which these findings are transferable. Third, the study relied on self-reported data, which may be subject to recall bias and social desirability. We also acknowledge that, although participants spontaneously reported in conversations that their avoidance of and isolation from others during the COVID-19 pandemic was motivated by the pandemic, our behavioral questions asked whether participants isolated themselves and avoided others *during* the pandemic (rather than explicitly *because* of the pandemic). Therefore, although unlikely, it is possible that some participants engaged in these behaviors for reasons unrelated to COVID-19.

Furthermore, the study did not include objective measures of COVID-19 infection or exposure, as post-factum verification would have been unreliable, and the region experienced a low incidence of COVID-19 (Asia Pacific Report, 2020). Additionally, we acknowledge potential order effects, as participants were asked about folk stories after answering questions regarding COVID-19. It is possible that the salience of the pandemic primed the retrieval of folk stories of human-to-human infectious diseases. While this may have increased the reported availability of these stories, it also highlights the cognitive accessibility of information about disease-related stories during times of crisis. Moreover, future research would benefit from employing more diverse methodological approaches. To better understand the cognitive mechanisms linking narrative content to health behaviors, researchers could utilize ethnographically relevant methods such as free-listing tasks (Purzycki, 2025). Such techniques would allow for a more precise mapping of the salient features of disease threats (e.g., how COVID-19 or other contagious diseases are conceptualized locally) and how these features overlap with the cognitive models embedded in folk stories. Future research could also employ longitudinal design, which would allow to track behavioral responses to disease outbreaks and how they are related to folk stories. Such design may be, however, difficult for various reasons to implement. An alternative may be experimental designs, which would permit stronger causal evidence by manipulating exposure to epidemic folk stories while holding other factors constant.

Fourth, this study did not examine the storytelling process itself, including the specific narrative content, performance contexts, or the role of audience in shaping and reinforcing behavioral norms. Future research should investigate how stories are transmitted, who tells them to whom, and how audience engagement influences the adoption of health-protective behaviors. Understanding these narrative dynamics would provide deeper insights into the mechanisms through which traditional knowledge translates into protective action.

Fifth, we acknowledge that our reductionist approach (i.e., analyzing only recommendations derived from folk stories rather than the stories in their entirety) may have resulted in the loss of important contextual information. Analyzing complete folk stories would have provided richer data and preserved narrative nuances relevant to understanding the transmission and impact of traditional epidemic knowledge. Sixth, inspection of the raw frequencies revealed that responses to the fear, avoidance, and distancing items were largely concentrated at extreme ends of the 5-point Likert scale. However, across all robustness checks and models’ specifications, the pattern of results was consistent with the main mediation analysis—the indirect effect of folk story knowledge on social distancing via fear remained significant, and the direct effect remained non-significant, providing further support that the strange distributional properties of the data are not the main reason for the observed associations.

Finally, the cross-sectional design of our study prevents us from drawing strong causal conclusions. While our mediation model suggests that story knowledge drives fear, it is also possible that individuals with higher baseline anxiety or fear are more likely to recall or seek out threatening stories (recall bias). Additionally, we did not collect information about potential confounding variables such as personality traits (e.g., neuroticism), socioeconomic status, or specific knowledge of public health initiatives. It remains possible that these unmeasured factors influence both the retention of folk story knowledge and compliance with safety measures. However, we note that observational designs can, in principle, yield useful causal evidence when their assumptions are stated explicitly and potential biases are assessed systematically (Rohrer, 2018). In this spirit, we have clearly articulated the causal target of inferences, identified one of the most plausible threats to identification, such as the potentially confounding role of dispositional anxiety or neuroticism, reverse causality in the relationship between story knowledge and fear, as well as measurement error in self-reported knowledge, and interpret our findings accordingly.

In conclusion, our research provides preliminary evidence that knowledge of Indigenous epidemic folk stories about human-to-human infectious diseases may be associated with higher levels of fear and greater social distancing during modern health crises. These findings are consistent with the hypothesis that storytelling may serve an adaptive function at the individual level, not by providing technical instruction manuals on what exactly to do, but by functioning as an alarm system. Specifically, the results of the mediation model suggest that narratives about ancestral outbreaks may be related to increased emotional vigilance (i.e., fear) and, in turn, greater behavioral avoidance (i.e., social distancing). However, our cross-sectional design, the exploratory nature of the mediation analysis, and the sample being limited to Asmat men in West Papua preclude strong or definitive causal inferences. Future research should employ experimental or longitudinal designs to test causal mechanisms, examine whether these patterns generalize across Indigenous communities and cultural contexts, and identify the narrative elements most likely to activate threat-related responses.

## Supplemental Material

sj-txt-1-evp-10.1177_14747049261439652 - Supplemental material for The Role of Indigenous Epidemic Folk Stories in Shaping Adaptive Disease-Related Behaviors in West Papua Supplemental material, sj-txt-1-evp-10.1177_14747049261439652 for The Role of Indigenous Epidemic Folk Stories in Shaping Adaptive Disease-Related Behaviors in West Papua by Kiriakos Chatzipentidis, Marta Kowal and Piotr Sorokowski in Evolutionary Psychology

sj-docx-2-evp-10.1177_14747049261439652 - Supplemental material for The Role of Indigenous Epidemic Folk Stories in Shaping Adaptive Disease-Related Behaviors in West Papua Supplemental material, sj-docx-2-evp-10.1177_14747049261439652 for The Role of Indigenous Epidemic Folk Stories in Shaping Adaptive Disease-Related Behaviors in West Papua by Kiriakos Chatzipentidis, Marta Kowal and Piotr Sorokowski in Evolutionary Psychology

sj-csv-3-evp-10.1177_14747049261439652 - Supplemental material for The Role of Indigenous Epidemic Folk Stories in Shaping Adaptive Disease-Related Behaviors in West Papua Supplemental material, sj-csv-3-evp-10.1177_14747049261439652 for The Role of Indigenous Epidemic Folk Stories in Shaping Adaptive Disease-Related Behaviors in West Papua by Kiriakos Chatzipentidis, Marta Kowal and Piotr Sorokowski in Evolutionary Psychology

sj-xlsx-4-evp-10.1177_14747049261439652 - Supplemental material for The Role of Indigenous Epidemic Folk Stories in Shaping Adaptive Disease-Related Behaviors in West Papua Supplemental material, sj-xlsx-4-evp-10.1177_14747049261439652 for The Role of Indigenous Epidemic Folk Stories in Shaping Adaptive Disease-Related Behaviors in West Papua by Kiriakos Chatzipentidis, Marta Kowal and Piotr Sorokowski in Evolutionary Psychology

sj-xlsx-5-evp-10.1177_14747049261439652 - Supplemental material for The Role of Indigenous Epidemic Folk Stories in Shaping Adaptive Disease-Related Behaviors in West Papua Supplemental material, sj-xlsx-5-evp-10.1177_14747049261439652 for The Role of Indigenous Epidemic Folk Stories in Shaping Adaptive Disease-Related Behaviors in West Papua by Kiriakos Chatzipentidis, Marta Kowal and Piotr Sorokowski in Evolutionary Psychology
