# Supplementary material for: The Role of Indigenous Epidemic Folk Stories in Shaping Adaptive Disease-Related Behaviors in West Papua
Source: Evol Psychol. 2026 Apr 28;24(2):14747049261439652. doi: 10.1177/14747049261439652 (PMC13145047; doi:10.1177/14747049261439652)
Supplement: sj-docx-2-evp-10.1177_14747049261439652 - Supplemental material for The Role of Indigenous Epidemic Folk Stories in Shaping Adaptive Disease-Related Behaviors in West Papua [file sj-docx-2-evp-10.1177_14747049261439652.docx]

**Supplementary Materials**

**Table S1**

Spearman correlation coefficients between the three variables of interest with data from participants who responded 3 to any of the two measures of social distancing (below the diagonal) and without data from these participants (above the diagonal).

|  | 1 | 2 | 3 |
| --- | --- | --- | --- |
| 1. Fear of COVID-19 | - | 0.559*** | 0.346** |
| 2. Social distancing | 0.558*** | - | 0.239* |
| 3. Folktale knowledge | 0.381*** | 0.244* | - |

*Note.* * *p* <.05, ** *p* < .01, *** *p* < .001.

**Table S2**

Results of the mediation model without data from participants who responded 3 to any of the two measures of social distancing.

| **Path** | **B** | **SE** | **95% CI** | ***p*** |
| --- | --- | --- | --- | --- |
| Folktale knowledge -> Fear of COVID-19 (path a) | 0.696 | 0.199 | [0.306,1.086] | <.001*** |
| Fear of COVID-19 -> Social distancing (path b) | 0.692 | 0.097 | [0.501,0.883] | <.001*** |
| Folktale knowledge -> Social distancing (direct effect) | 0.027 | 0.185 | [-0.335,0.389] | .884 |
| Indirect effect (path a* b) | 0.482 | 0.153 | [0.181,0.782] | .002** |
| Folktale knowledge -> Social distancing (total effect) | 0.509 | 0.22 | [0.077,0.94] | .021* |

*Note.* * *p* <.05, ** *p* < .01, *** *p* < .001.

**Table S3**

Spearman correlation coefficients between the four variables of interest (full sample).

|  | **1** | **2** | **3** |
| --- | --- | --- | --- |
| 1. Fear of COVID-19 | - |  |  |
| 2. Social distancing | 0.558*** | - |  |
| 3. Folktale knowledge | 0.381*** | 0.244* | - |
| 4. Age | 0.114 | 0.137 | 0.113 |

*Note.* * *p* <.05, ** *p* < .01, *** *p* < .001.

**Table S4**

Results of the mediation model controlling for Age.

| **Path** | **B** | **SE** | **95% CI** | ***p*** |
| --- | --- | --- | --- | --- |
| Folk story knowledge -> Fear of COVID-19 (path a) | 0.703 | 0.184 | [0.343,1.062] | <.001*** |
| Fear of COVID-19 -> Social distancing (path b) | 0.686 | 0.091 | [0.508,0.865] | <.001*** |
| Age -> Social distancing | 0.012 | 0.010 | [-0.007,0.032] | .208 |
| Age -> Fear of COVID-19 | -0.004 | 0.012 | [-0.027,0.019] | .731 |
| Folk story knowledge -> Social distancing (direct effect) | -0.028 | 0.169 | [-0.359,0.303] | .869 |
| Indirect effect (path a* b) | 0.482 | 0.141 | [0.759,0.482] | <.001*** |
| Folk story knowledge -> Social distancing (total effect) | 0.454 | 0.201 | [0.848,0.454] | .024* |

*Note.* * *p* <.05, ** *p* < .01, *** *p* < .001.

**Table S5**

Results of the mediation model controlling for the Village (factor with 4 levels).

| **Path** | **B** | **SE** | **95% CI** | ***p*** |
| --- | --- | --- | --- | --- |
| Folk story knowledge -> Fear of COVID-19 (path a) | 0.701 | 0.165 | [0.377,1.024] | <.001*** |
| Fear of COVID-19 -> Social distancing (path b) | 0.828 | 0.094 | [0.644,1.012] | <.001*** |
| Village -> Social distancing | 0.493 | 0.133 | [0.233,0.753] | <.001*** |
| Village -> Fear of COVID-19 | -0.589 | 0.138 | [-0.858,-0.319] | <.001*** |
| Folk story knowledge -> Social distancing (direct effect) | -0.101 | 0.159 | [-0.413,0.21] | .523 |
| Indirect effect (path a* b) | 0.58 | 0.152 | [0.877,0.58] | <.001*** |
| Folk story knowledge -> Social distancing (total effect) | 0.478 | 0.199 | [0.869,0.478] | .016* |

*Note.* * *p* <.05, ** *p* < .01, *** *p* < .001.

**Table S6**

A summary of the mediation analysis with variables of interest treated as categorical variables (Avoiding others as Y).

| **Path** | **B** | **SE** | **95% CI** | ***p*** |
| --- | --- | --- | --- | --- |
| Folk story knowledge -> Fear of COVID-19 (path a) | 1.156 | 0.374 | [0.423,1.888] | .002** |
| Fear of COVID-19 -> Avoiding others (path b) | 0.823 | 0.07 | [0.686,0.961] | <.001*** |
| Folk story knowledge -> Avoiding others (direct effect) | -0.124 | 0.194 | [-0.504,0.257] | .525 |
| Indirect effect (path a* b) | 0.952 | 0.31 | [1.56,0.952] | .002** |
| Folk story knowledge -> Avoiding others (total effect) | 0.828 | 0.356 | [1.526,0.828] | .020* |

*Note.* * *p* <.05, ** *p* < .01, *** *p* < .001.

**Table S7**

A summary of the mediation analysis with variables of interest treated as categorical variables (Keeping distance as Y).

| **Path** | **B** | **SE** | **95% CI** | ***p*** |
| --- | --- | --- | --- | --- |
| Folk story knowledge -> Fear of COVID-19 (path a) | 1.156 | 0.374 | [0.423,1.888] | .002** |
| Fear of COVID-19 -> Keeping distance (path b) | 0.688 | 0.117 | [0.459,0.917] | <.001*** |
| Folk story knowledge -> Keeping distance (direct effect) | 0.06 | 0.22 | [-0.371,0.491] | .785 |
| Indirect effect (path a* b) | 0.795 | 0.283 | [1.349,0.795] | .005** |
| Folk story knowledge -> Keeping distance (total effect) | 0.855 | 0.344 | [1.53,0.855] | .013* |

*Note.* * *p* <.05, ** *p* < .01, *** *p* < .001.
